# A Sub-30 mpH Resolution Thin Film Transistor-Based Nanoribbon Biosensing Platform

**DOI:** 10.3390/s17092000

**Published:** 2017-09-01

**Authors:** Ioannis Zeimpekis, Konstantinos I. Papadimitriou, Kai Sun, Chunxiao Hu, Peter Ashburn, Hywel Morgan, Themistoklis Prodromakis

**Affiliations:** Nanoelectronics & Nanotechnology Research Group, Electronics and Computer Science, University of Southampton, Southampton SO17 1BJ, UK; izk07r@ecs.soton.ac.uk (I.Z.); ks5@ecs.soton.ac.uk (K.S.); Chunxiao.Hu@glasgow.ac.uk (C.H.); hm@ecs.soton.ac.uk (H.M.); t.prodromakis@soton.ac.uk (T.P.)

**Keywords:** analog-to-digital conversion, biosensor, nanoribbon, pH sensing, Point-of-Care diagnostics, switched capacitor, TFT, urea-urease reaction

## Abstract

We present a complete biosensing system that comprises a Thin Film Transistor (TFT)-based nanoribbon biosensor and a low noise, high-performance bioinstrumentation platform, capable of detecting sub-30 mpH unit changes, validated by an enzymatic biochemical reaction. The nanoribbon biosensor was fabricated top-down with an ultra-thin (15 nm) polysilicon semiconducting channel that offers excellent sensitivity to surface potential changes. The sensor is coupled to an integrated circuit (IC), which combines dual switched-capacitor integrators with high precision analog-to-digital converters (ADCs). Throughout this work, we employed both conventional pH buffer measurements as well as urea-urease enzymatic reactions for benchmarking the overall performance of the system. The measured results from the urea-urease reaction demonstrate that the system can detect urea in concentrations as low as 25 μM, which translates to a change of 27 mpH, according to our initial pH characterisation measurements. The attained accuracy and resolution of our system as well as its low-cost manufacturability, high processing speed and portability make it a competitive solution for applications requiring rapid and accurate results at remote locations; a necessity for Point-of-Care (POC) diagnostic platforms.

## 1. Introduction

Measurement of enzyme-substrate reactions underpin many sensing technologies, including the Enzyme-Linked Immuno Sorbent Assays (ELISA). Whether they take place in a clinical environment using highly sophisticated clinical instruments or in a POC setting, the core principles of the reactions’ detection are similar, however, they differ in the speed of the assessment and its overall accuracy. Ideally, POC diagnostic tools require fast and accurate detection methods delivered via affordable manufacturing techniques without compromising the compactness of the device. Currently, a plethora of methods are being used for the detection of enzyme-substrate-based assays, including colorimetric, fluorometric, chemiluminescent and complex biochemical labelling methods [1,2,3,4]. However, the vast majority requires bulky and expensive optical or electronic equipment for the read-out process [5], therefore, they are not appropriate for POC applications. A trade-off between size, accuracy and speed is therefore inevitable and remains an issue for compact POC diagnostic tools. Modern electronics offer mature circuit design techniques and are capable of providing sufficient measurement accuracy and versatility in signal processing which can be linked via high-speed data transmission. What is even more interesting is that all the aforementioned properties can be accommodated into a single mixed-signal microchip with minute power demands, enabling use in remote locations without jeopardizing the overall performance. The bottleneck for delivering reliable and cost-effective POC diagnosis still remains in the sensing element capabilities, its integration with the read-out electronics and the accuracy and specificity of the biological assay.

Due to the fact that many enzymatic reactions modify the pH of the solution, a widely used approach includes the detection via electrochemical means with Ion Sensitive Field Effect Transistors (ISFETs) [4]. ISFETs implemented in commercially available Complementary Metal-Oxide-Semiconductor (CMOS) processes have provided affordable and power-efficient POC solutions to (bio)chemical monitoring. Assays that have been monitored using ISFETs include DNA and protein detection [6,7,8,9,10,11,12]. Despite their capabilities, ISFETs are plagued by long-term drift, hysteresis, thermal drift, mismatches and large referred threshold voltages [13]. These issues have a direct impact upon the detection limits and the sensor’s reliability. To address these challenges, a large amount of work has been carried out to improve their performance [14,15,16], however, most of these improvements lead to either expensive or challenging fabrication processes that, on the majority, may not be suitable for POC diagnostic tools.

In recent years, silicon nanowires and nanoribbons have been employed as sensors in an attempt to improve on ISFET biosensors. These devices offer improved sensitivity without the need for complicated device architectures [17,18,19,20,21]. Early approaches used bottom-up technologies to fabricate nanowires but top-down technologies are generally preferred, due to ease of manufacturing as they provide precise dimension and placement control, while offering a route to manufacturing. The display industry has demonstrated that high quality and high yield devices can be manufactured using TFT technologies and lately these technologies have been adopted for the manufacturing of low cost sensors [17,18]. A number of publications have verified the potentials of TFT nanoribbons as pH and protein sensors [19,20,21], but none of them combined a TFT biosensor with a portable, low-cost read-out platform. In our previous work [21] we demonstrated the detection of C-reactive protein (CRP) in human serum via a miniature bead-based ELISA. The TFT nanoribbon sensor was able to measure the reaction products from the ELISA via pH changes. For the purposes of that experiment, the TFT nanoribbon sensor was coupled to a Keithley 6482 picoammeter/voltage source, which provided the required biasing voltages to the TFT sensor and the read-out for the measurements. However, it is an important requirement for POC diagnostics that such an assay can be performed by a miniaturized, portable, embedded system, capable of measuring different concentrations of reaction products precisely, in high-resolution and with low noise.

Herein, we progress our previous research and demonstrate a complete, state-of-the-art bioelectronic system composed of a thin film biosensor and a high-resolution, low-noise, portable read-out circuit, which exploits the switched-capacitor integration technique. In order to investigate the full potential and capabilities of this combination, we employ a well-known substrate-enzymatic reaction to produce small pH changes on the surface of our biosensor. The system is able to measure minute pH changes in real-time, with a very low noise level equivalent to 10 mpH. The ultra-high sensitivity of the system provides a promising solution for high sensitivity measurement of enzyme-substrate reactions and for ELISA-based POC diagnostics, offering small size, accuracy, and speed, without the need to drastically increase the cost.

## 2. Materials and Methods

### 2.1. The TFT Nanoribbon Fabrication

The fabrication of the sensors was based on an improved version of our top-down TFT technology [17]. The wafers used were 6-inch p-type wafers with a resistivity of 1–30 Ohm x cm. The first step of the fabrication was the growth of the bottom dielectric stack. This was achieved by wet thermal oxidation at 1000 °C to grow 100 nm of SiO_2_ followed by the deposition of a 300 nm Si_3_N_4_ layer, using low pressure chemical vapour deposition (LPCVD) at 800 °C. A 20 nm in-situ phosphorous doped amorphous silicon (a-Si) layer was then deposited through plasma enhanced chemical vapor deposition (PECVD) at 200 °C and patterned to form the ribbons on top of the dielectric stack. The wafer was then annealed at 900 °C in oxygen. In this process, the a-Si was re-crystalized into polysilicon and thinned down to 15 nm. The oxidation left an 8.5 nm SiO_2_ layer which serves as the first dielectric of the top-gate dielectric stack. A 17 nm Al_2_O_3_ layer was then grown as the second dielectric of the top dielectric stack, using thermal atomic layer deposition (ALD) at 200 °C. The metal layer pattern was subsequently transferred on the wafer by a negative photoresist. A stack of 20 nm Ti, 200 nm TiN, 20 nm Ti, 750 nm Ag was then sputtered and patterned using lift-off. SU-8 3005 was used to form a 5 μm passivation layer to protect the metal contacts from shorting in the liquid. Next, the exposed silver layer was chlorinated in pure bleach for 1 min to form an on-chip Ag/AgCl reference electrode. Finally, a 3 mm thick PMMA layer that was previously patterned with a laser cutter was stuck onto the surface with double-sided 3 M tape to form 50 μL wells.

### 2.2. The Bioinstrumentation Board

**Figure A1:**
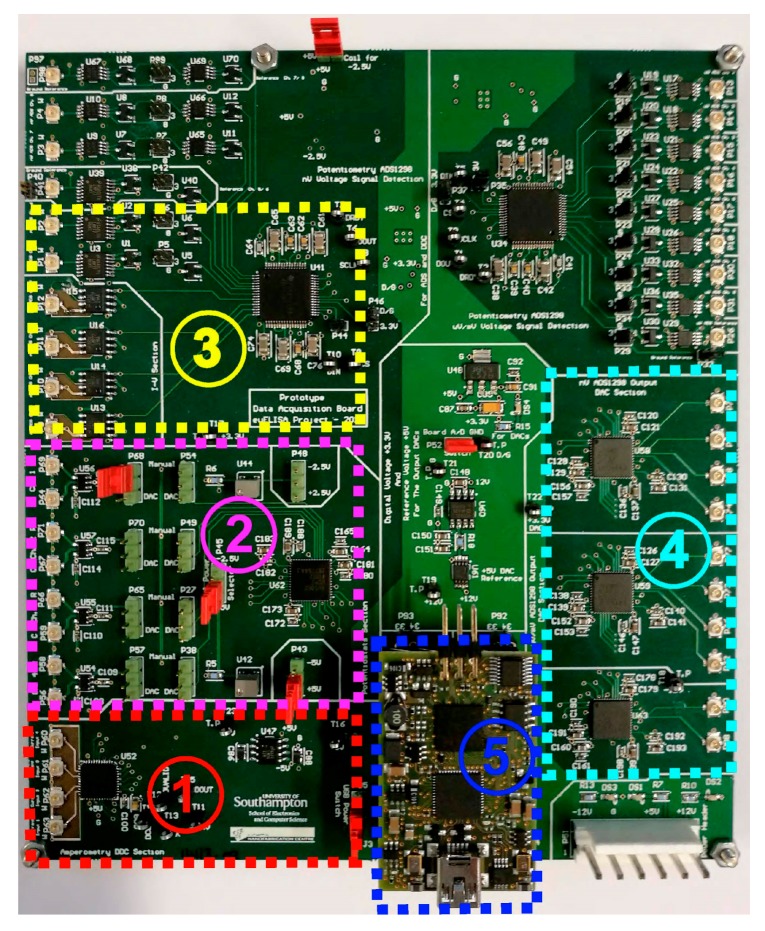
The proposed, fabricated bioinstrumentation board with its five distinct compartments. (1) Dual switched-capacitor integrator section; (2) DAC section providing the appropriate biasing voltages to the TFT sensor; (3) Standard transimpedance amplifier configuration for the preliminary characterisation of the setup; (4) Auxiliary DAC section for sensor biasing and analogue data output; (5) FPGA-based DSP unit.

**Figure A2:**
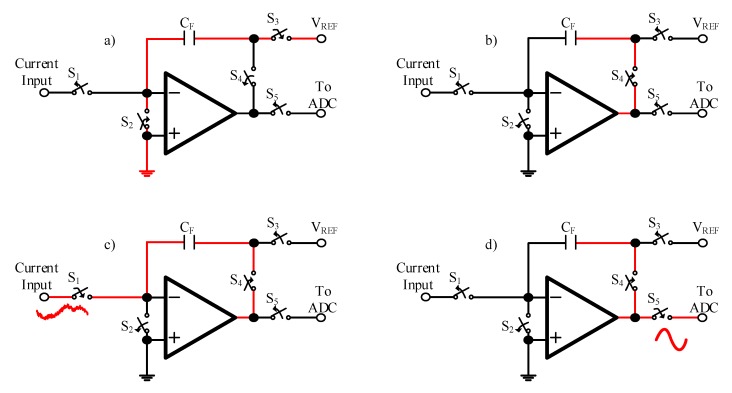
Schematics of the DDC switched-capacitor integrator configurations. (**a**) **Reset configuration**: the capacitor C_F_ is charged to the reference voltage at the completion of an A/D conversion; (**b**) **Wait configuration**: at this stage the circuit is waiting before it enters the integration stage; (**c**) **Integrate configuration**: the input current is charging the capacitor C_F_ for a time period defined by the user, appropriate for the input current range; (**d**) **Convert configuration**: discharge phase of the capacitor C_F_, which results to an output voltage analogous to the input current that charged the capacitor during the integration stage. This voltage directly “feeds” the input of the 20-bit ADCs.

A custom-made, 6-layer Printed Circuit Board (PCB) has been designed and fabricated for the detection of minute input currents and the appropriate biasing of the TFT sensor (see Figure A1 in Appendix A). The dual switched-capacitor integrator circuit topology used for the nanoribbon current sensing was part of a commercially available IC component manufactured by Texas Instruments. The 4-channel DDC family current-input ADC includes these dual integrators combined with high-resolution ΔΣ modulators. The total current supply of the specific IC does not exceed 15 mA, making it suitable for battery-powered operation [22]. The switched capacitor integrator is an alternative topology to the traditional current-to-voltage conversion technique using a transimpedance amplifier (TIA). In a switched capacitor circuitry, the input biosensor current charges a capacitor, instead of passing through the usually high value feedback resistor of the commonly used TIA. Since the switched-capacitor technique does not require an ohmic element, the input biosensor current is not distorted by the strong presence of thermal noise that accompanies the input resistor. Therefore, the noise properties of the integrator circuitry are significantly better when compared to a TIA [22]. Moreover, the fact that this IC includes two symmetrical integrator branches, allows for the continuous monitoring of the input signal. While one branch is integrating the input current by charging its integration capacitor, the complementary circuitry in the other branch is discharging its integration capacitor, sending its output voltage to the chip’s ADC and *vice versa*. A schematic of the switched capacitor integration stages can be seen in the Appendix A, Figure A2.

Once the capacitor’s output has been sent to the ΔΣ modulators, it converts into digital format, allowing further processing without introducing any additional noise to the system. The immediate digitisation of the analogue signal protects the measurement data from further noise and therefore, provides a higher signal-to-noise ratio (SNR), when compared to the aforementioned traditional techniques. The digital data is subsequently sent to a commercially available FPGA-based Digital Signal Processing (DSP) unit, which allows the data to be sent directly to the user’s PC or to be converted to an analogue voltage signal using a Digital-to-Analog Converter (DAC). The FPGA that was selected in this case was a Spartan3e from Xilinx. The FPGA was clocked from a 48 MHz clock module and was programmed via a USB 2.0 interface. Finally, the fabricated bioinstrumentation platform has a large number of auxiliary circuitry, able to provide constant, high precision biasing voltages ranging from −12 V up to +12 V. These can be used to bias more complicated biosensor topologies such as dual-gate or differential ones and effectively removes the need for external biasing circuits.

### 2.3. Interface of Biosensor with the Bioinstrumentation Platform

**Figure A3:**
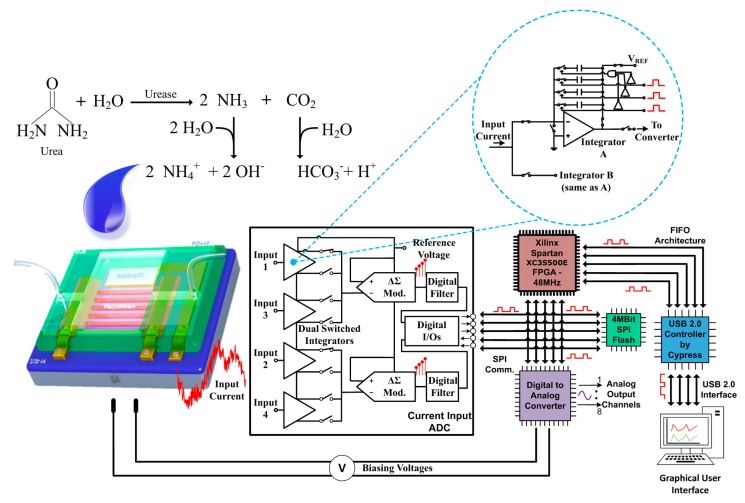
Schematic representation of the complete measurement setup.

Figure A3 in Appendix A shows a schematic representation of the complete measurement system. The sensor was bonded with silver conductive epoxy and wire-bonded with a wedge aluminum wire wire-bonder (Delvotec 5430, F&K Delvotec Bondtechnik GmbH, Germany) onto a PCB. The PCB was enclosed in a grounded aluminum connection box that served as an electrical and light shield. Although the bioinstrumentation board does not require any additional equipment in order to power, bias and read-out the TFT sensor, for these experiments we have used it in combination with the Agilent B1500A (Agilent Technologies Singapore (International) Pte. Ltd., Singapore) semiconductor analyser. The reason lies within the verification of the measurements. This way we were able to obtain a low-noise record of the input current, in-sync with the data exported from the measurement system. The simultaneous collection of both the input and output currents of the bioinstrumentation board allowed us to consistently evaluate the noise performance of the electronic system.

The drain potential was kept at 100 mV whereas the reference electrode was biased at −700 mV throughout the experiments. During the Id-Vlg measurements the semiconductor analyser was used to sweep the reference electrode between −1.3 and −0.3 V. The source of the sensor was connected to the switched-capacitor read-out circuit, where the current was read and then sent to the parameter analyzer for storage. This setup allows the concurrent display of results from all the terminals, including that of the measurement board on the parametric analyser.

Measurements of pH were carried out using universal buffer solutions (0.1 M NaCl, 0.01 M citric acid, 0.01 M phosphoric acid and 0.02 M boric acid adjusted to pH values ranging from 3 to 9 by titration with a 1 M NaOH solution). The resolution and accuracy of the pH meter used was 10 mpH and 20 mpH, respectively, and the titration was performed with a precision at the second decimal point. The solutions were pipetted onto the sensor surface with the nanoribbons thoroughly washed with de-ionized water between measurements.

A well-known substrate-enzyme reaction was demonstrated using urea (Sigma) and urease (Sigma). Seven different concentrations of urea were prepared in a buffer (0.01 × PBS + 150 mM NaCl). Prior to each measurement, the entire sensing area (including the Ag/AgCl reference electrode) was incubated in 3% bovine serum albumin (BSA) in PBS to minimise non-specific binding. Urea solution (30 μL at various concentrations) was pipetted into the sensing window and the source-drain current was monitored against time. Subsequently, urease (20 μL at 0.45 mg/mL) was pipetted. Urease catalyses the hydrolysis of urea to ammonia and carbon dioxide leading to an increase in the pH as the reaction proceeds. This results in a decrease in the source-drain current (for n-type nanoribbon sensors), which was read by the bioinstrumentation board.

## 3. Results

### 3.1. TFT Biosensor Characterisation

**Figure 1:**
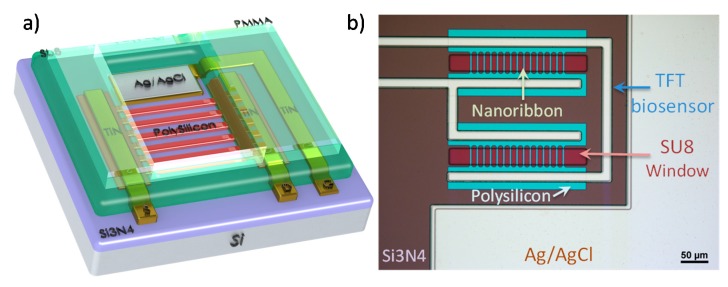
(**a**) Three-dimensional representation of the TFT nanoribbon biosensor; (**b**) Microscope picture of the TFT nanoribbon biosensor.

Figure 1a is a schematic representation of the ultra-thin TFT polysilicon nanoribbon biosensor. The sensor is fabricated using a three-mask TFT process [17] that uses an in-situ doped polysilicon layer, without highly doped source/drain regions. This work uses an improved polysilicon layer, compared to the previous work [17], that is additionally thinned down to 15 nm, offering higher sensitivity [23]. The 30 nanoribbons that comprise the sensor are 2 μm wide and are passivated by a SiO_2_/Al_2_O_3_ gate dielectric stack, optimised to enhance the sensitivity of the sensor [23]. The analyte confined to the sensor with an SU-8 window, which is 30 μm long. The sensor incorporates an integrated Ag/AgCl reference electrode that forms a liquid gate terminal with the analyte. A laser cut 3 mm thick PMMA layer is used to create a 50 μL well on top of the sensor. Figure 1b is a microscope picture of the actual sensor, showing the SU-8 passivation of the nanoribbons and the Ag/AgCl that is exposed to the analyte.

**Figure 2:**
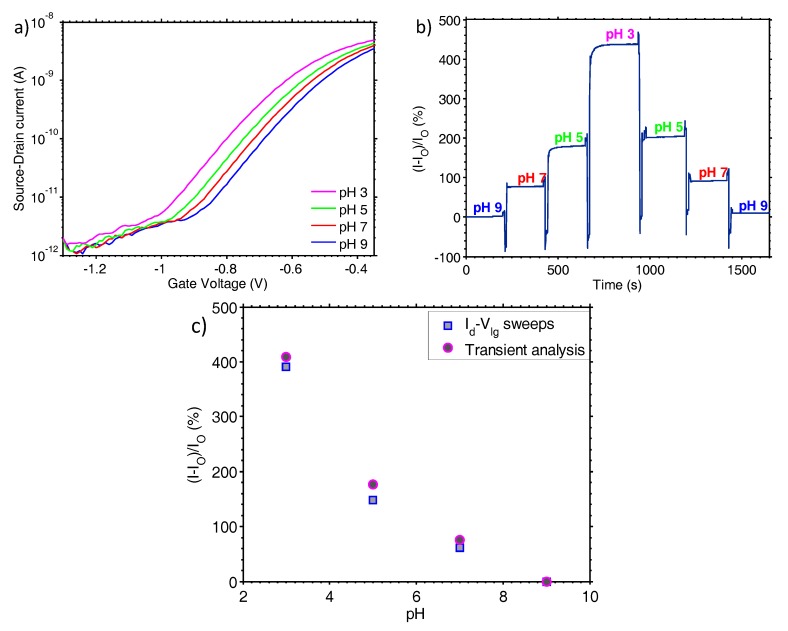
(**a**) Id-Vlg characteristic curves of the nanoribbon biosensor for a range of pH 3 to pH 9; (**b**) Normalised percentage drain current change of the biosensor for a range of pH from 3 to pH 9; (**c**) Nanoribbon biosensor pH sensitivity extracted from the Id-Vlg characteristic curves and transient measurements.

As a first calibration step, the biosensor was characterised using universal buffer solutions, ranging in pH from 3 to 9. Figure 2a shows representative results where the drain current is monitored by sweeping the liquid-gate from −1.3 to −0.45 V (Id-Vlg sweep) for pH values of 3, 5, 7 and 9. When the value of pH is increased, the Id-Vlg characteristic curve shifts to more positive voltage values. This is because a higher pH induces a negative surface charge on the sensor, which depletes the majority carriers (electrons) in the n-type TFT channel. The Id-Vlg characteristic curves for the four different buffers are parallel in the subthreshold region. Hence, monitoring the current change at a reference liquid gate voltage enables direct measurement of pH.

The sensor was selected to operate in the subthreshold region and consequently there is an exponential relation between the drain current and the liquid-gate voltage, for a fixed drain to source voltage (Vds). In our case, the subthreshold slope was extracted from the Id-Vlg characterisation data and found to be 150 mV/dec, indicating the excellent performance of the in-situ doped and thermally annealed polysilicon semiconductor. The operating point of the sensor lies in the middle of the subthreshold region, hence, for any fixed liquid gate bias measurement a −700 mV potential was used. The pH measurement was repeated for the same fixed liquid gate bias and the drain current levels for different pH values monitored with respect to time. Figure 2b shows the normalised drain current percentage change over time for pH 3 to pH 9 (calculated as the current at the pH of interest minus the current at pH 9, all divided by the current at pH 9 and then multiplied by 100). The transient response of the sensor demonstrates minimal hysteresis and drift, which is of paramount importance when aiming to detect minute pH changes. In Figure 2c, the current sensitivity of the sensor for different pH changes is shown, extracted from the Id-Vlg characteristic curves and transient measurements. The results from the two measurements demonstrate very similar trends and values, validating the robustness of both measurements. The overall sensitivity for a range of pH from 3 to 9 has been found equal to 436%. This translates to a sensitivity of 25.5%/pH, calculated based on the method shown in [23]. These pH results were used in the following experiments as calibration data for converting the current output changes from the switched-capacitor circuitry to pH changes.

### 3.2. Urea-Urease Enzymatic Reaction

**Figure 3:**
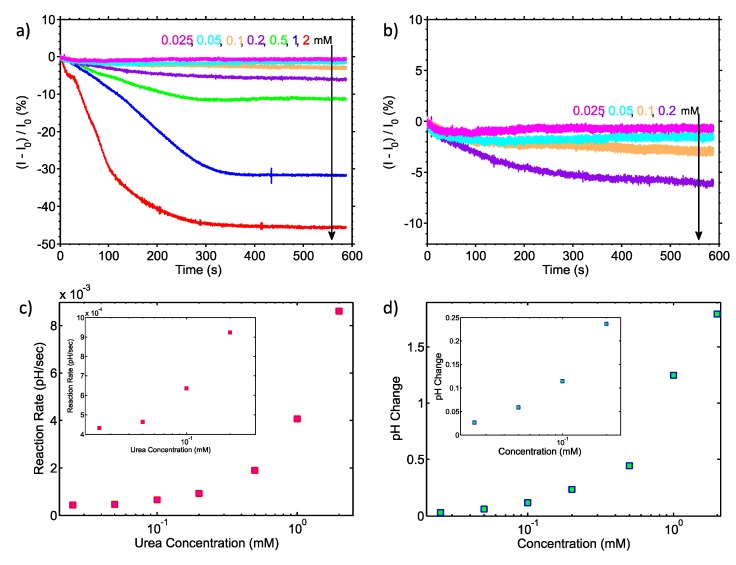
(**a**) Normalised drain current change with respect to time for different concentrations of urea; (**b**) Normalised drain current change for lower concentrations of urea; (**c**) Reaction rate vs urea concentrations; (**d**) Final extracted value of pH change as a function of urea concentration.

After characterisation, the sensor was tested by monitoring the urea-urease reaction. Figure 3a,b illustrate the transient results for seven different urea concentrations and a “zoomed-in” transient view of the lowest four concentrations, respectively. The assay was performed using 2 mM, 1 mM, 0.5 mM, and 0.2 mM of urea and the responses were 45.7%, 31.8%, 11.3% and 6%, respectively. Urea solutions of lower concentrations, i.e., 0.1, 0.05 and 0.025 mM were subsequently introduced onto the sensor, generating output changes of 2.9%, 1.5% and 0.68%, respectively. The drift rate was constant and equal for all measurements and was found to be approximately 2 pA/s, which is equivalent to 847 μpH/s, highlighting the stable operation of our sensor, as predicted by our initial characterisation measurements. It must be noted that each measurement was taken after an initial stabilisation of only 6 min (the overall measurement time, including stabilisation, was 15 min) hence the drift rate presented here is the “worst case” one and does not reflect long measurements or measurements after a long stabilisation step.

Figure 3c shows the reaction rate extracted from the results presented in Figure 3a,b, demonstrating that the reaction rate increases with increasing concentration, as expected. Moreover, Figure 3d shows the endpoint of the reactions, extracted from the results of Figure 3a and then converted to an equivalent pH change using the calibration data of Figure 2c. These data show that the system is capable of measuring minute pH changes, with the smallest concentration of urea (0.025 mM) giving a 27 mpH change.

**Figure 4:**
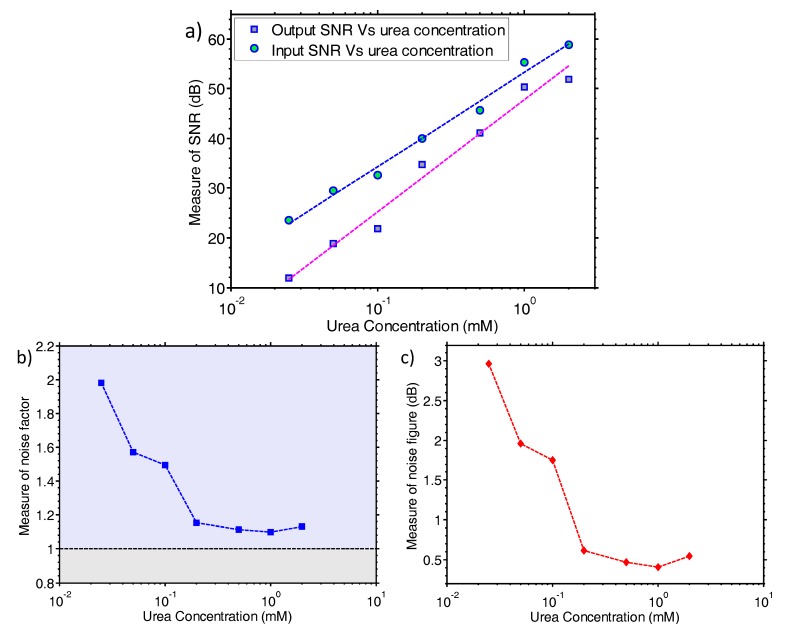
Noise analysis of the proposed system. (**a**) System input and output measure of SNR plotted against urea concentrations; (**b**) Measure of noise factor of the system vs urea concentrations; (**c**) Measure of noise figure of the system vs urea concentrations.

Finally, in order to investigate the noise properties of the system, noise analysis was performed on the collected sensor data. Figure 4a shows a measure of SNR for the input and output signals of the bioinstrumentation platform, plotted as a function of the urea concentrations. Moreover, Figure 4b,c are providing measures of the noise factor and noise figure of the system, respectively, all plotted as a function of the urea concentration. All previous noise analysis results were produced using a custom-made algorithm in Matlab^®^ [22] that divides the recorded signal into equal segments and computes the mean and the standard deviation in each segment for the recorded reactions. The whole measurement for each urea concentration was roughly 560 s, as it can be seen in Figure 3a, using a sampling rate of 10 samples per second. The standard deviation values provide an estimate of the noise amplitude “A_NOISE_” in time.

This algorithm can be considered as an indicative, “continuous-time” measurement of the system’s noise properties. A linear relationship between urea concentration and SNR has been found, allowing the system performance and limitations to be evaluated. As expected, the SNR values of the sensor input current signal are higher than those of the output signal. The SNR of the system is low for the low concentrations, highlighting the effect of the noise on the sensor’s signal. While the urea concentration is increasing and consequently the sensor’s signal is increasing as well, the SNR of the system increases in a linear fashion. In addition, Figure 4b,c demonstrate the trend of the noise figure and noise factor as the urea concentration increases and consequently the sensor’s signal is increasing as well. The stronger the signal is, the closer the noise factor (SNR_IN_/SNR_OUT_) approaches unity. By using the detected noise of the system as a reference point, the absolute limit of detection (LoD) of the whole system can be extrapolated. Based on the results provided by our custom algorithm, the inherent noise of the system is an approximate 0.4% change, leading to an estimated 10 mpH LoD.

## 4. Discussion

The Id-Vlg measurements of Figure 2a reveal that the TFT nanoribbon biosensor has an excellent subthreshold slope of 150 mV/dec. This was achieved by fabricating the semiconducting channel from a low-doped ultra-thin polysilicon layer that offers a very small depletion capacitance. The steep subthreshold slope is also attributed to the high gate dielectric capacitance, and low interface traps between the channel and the gate dielectric. By using the sensor in the subthreshold region, we effectively employ it as a “non-linear amplifier”. As shown in [23], a steeper value of subthreshold slope leads to a higher value of current sensitivity.

The sensitivity of the sensor to pH changes was confirmed both via Id-Vlg measurements and by monitoring the transient response, with the results from the two measurements deviating by a maximum of 22% at pH 7. This deviation is attributed to the non-continuous state in which the Id-Vlg measurements are performed and implies that there is a small amount of drift and hysteresis associated with the sensor measurement. Therefore, although the sensor has a high resolution, its precision is compromised by drift and hysteresis. The drift can be improved by further optimisation of the dielectric and electronic compensation, whereas the nature of hysteresis will have to be determined and be compensated through the measurement protocol. For pH 7 and pH 5, the hysteresis is found to be 18% and 13%, respectively, whereas the maximum drift at pH 5 is 2.9% per 100 s but as low as 0.4% per 100 s at pH 9. The low drift rate is achieved due to the high quality gate dielectric stack. The top-gate dielectric is composed of 8.5 nm of thermally grown SiO_2_ and 17 nm of atomic layer deposited Al_2_O_3_. The SiO_2_ provides a low interface state density between the channel and the dielectric, whereas the Al_2_O_3_ provides a robust hydration interface during liquid measurements. As atomic layer deposited Al_2_O_3_ is highly impermeable to ions when compared to SiO_2_, the drift due to interactions with free ions in the measurement liquid is minimal.

The urea-urease was used to characterise the sensor’s pH resolution. The reaction endpoint pH and rates were extracted from all seven substrate concentrations in the mM and μΜ range. The endpoint was defined as the pH value change after 560 s averaged for a period of 5 s at a 100 ms time interval from the introduction of the enzyme. As predicted by the Michaelis-Menten kinetics model [24], when the concentration of the substrate is much lower than the Michaelis constant K_m_ (the substrate concentration at which the reaction rate is half of the maximum), the relation between reaction rate and concentration is linear. In our previous work we calculated K_m_ to be 15.9 mM [21] and since the concentrations of urea used in this work are less than 2 mM both reaction rate and endpoint value methods show a linear relation between concentration and current change.

The results shown in Figure 3 indicate that our system is capable of reading urea concentrations as low as 25 μM. Using the calibration data from the continuous time measurement of Figure 2 and assuming that small pH changes may be considered to result in a linear change of the biosensor current [23], the endpoint of the 25 μM urea substrate is equivalent to a change of 27 mpH. This result shows that our embedded system approaches the theoretical limit of 10 mpH for nanoribbon biosensors, as demonstrated in [25,26], without resorting to any additional filtering or complex read-out equipment. It also verifies the statement in [25] where a large area sensor is predicted to have a better LoD than an aggressively scaled device (i.e., nanoribbon vs nanowire sensor). Regarding the noise properties of the proposed system, as can be seen from Figure 3 and particularly in Figure 4, the noise level is low enough to enable urea measurements to concentrations down to 25 μM. The electronic system records the enzyme reaction in real-time and “*projects*” the data back to the user directly, without the need for any further analogue or digital filtering stage.

**Figure 5:**
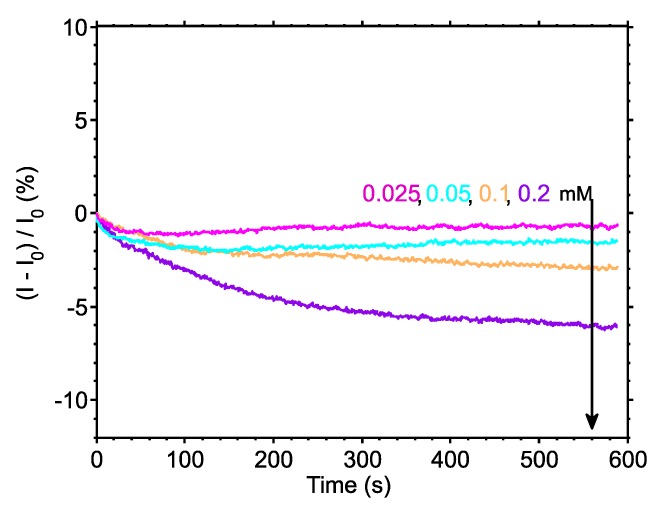
Filtered transient results of Figure 3b. The applied digital filter clearly allows us to increase our system’s resolution even further.

To demonstrate how filtering can increase the resolution of our system, we used a low-pass filter on the results of Figure 3b and plotted the filtered output in Figure 5. The filter applied was a 10th order (number of coefficients-1) finite impulse response (FIR) low-pass digital filter, with cut-off frequency at 0.5 Hz. Similar types of standard digital filters can be implemented on the processing unit of the proposed bioinstrumentation platform, without compromising the overall processing speed and consequently the system’s response. As expected, the noise levels of all signals have been reduced significantly, allowing us to clearly distinguish between all four low urea concentrations. The flexibility and versatility of the proposed system, with respect to data acquisition and data processing, allows the user to select whether or not the data should be processed, depending on the sensitivity and signal range of their experiment. The ability to process the incoming sensor data “on the fly” allows for a significant increase in the system’s resolution, as shown in Figure 5. As a result, the proposed biosensing setup becomes capable of performing in even more demanding experiments.

## 5. Conclusions

In this work, a new embedded system for biosensing is proposed for ultra-low pH measurements, comprised of an ultra-thin 15 nm TFT nanoribbon and a current-input ADC, which exploits the dual switched-capacitor architecture. The combination of these two elements allowed us to detect very low pH changes down to 27 mpH, by employing the urea-urease enzymatic reaction. The obtained results highlighted the capabilities of the system, taking into consideration that the obtained signal has not been filtered or post-processed by any possible analogue or DSP mean. In addition, we also demonstrated that the noise levels could be significantly reduced to improve the measurement resolution by implementing a standard digital filter through our processing unit. All the above, combined with the fact that the proposed device is portable and has very small power requirements, makes it an excellent candidate for POC diagnostic applications.

Herein we have demonstrated for the first time that the TFT nanoribbon sensor combined with a high-resolution switched capacitor IC allowed us to come very close to the theoretical limit of 10 mpH. This implies that further optimisation, especially in the microfluidics part of the system, will allow us to proceed reliably with even more demanding applications, where minute pH or charge changes are imperative.
